# Mobile device use among emergency department healthcare professionals: prevalence, utilization and attitudes

**DOI:** 10.1038/s41598-021-81278-5

**Published:** 2021-01-21

**Authors:** Eveline Hitti, Dima Hadid, Jad Melki, Rima Kaddoura, Mohamad Alameddine

**Affiliations:** 1 Department of Emergency Medicine, American University of Beirut, Beirut, Lebanon; 2 Department of Communication Arts, Lebanese American University, Beirut, Lebanon; 3 Department of Health Management and Policy, American University of Beirut, PO Box 110236, Riad El Solh, Beirut, 1107-2020 Lebanon; 4 College of Medicine, Mohammed Bin Rashid University of Medicine and Health Sciences, Dubai, UAE

**Keywords:** Health policy, Health services, Public health

## Abstract

Mobile devices are increasingly permeating healthcare and are being regularly used by healthcare providers. We examined the prevalence and frequency of mobile device use, and perceptions around clinical and personal usage, among healthcare providers (attending physicians, residents, and nurses) in the Emergency Department (ED) of a large academic medical center in Lebanon. Half of the target population (N = 236) completed the cross-sectional electronic questionnaire. Mobile device usage for personal matters was uniform across all providers, with the highest usage reported by medical students (81.3%) and lowest by attendings (75.0%). Medical formulary/drug referencing applications were the most common application used by providers followed by disease diagnosis/management applications, 84.4% and 69.5% respectively. Most respondents agreed that mobile devices enabled better-coordinated care among providers and were beneficial to patient care. Most respondents also agreed that mobile device use assisted in quickly resolving personal issues and reduced their feeling of stress, yet the majority did not feel that personal usage improved performance at work. Study findings revealed that although healthcare providers value mobile devices’ positive impact on coordination of care, the reverse spillover effect of personal issues into the workplace enabled by mobile devices might have some negative impact on performance of staff at work.

## Introduction

Mobile devices increasingly permeate the healthcare workplace and their regular use by healthcare providers (HCPs) is on the rise^1–3^. The impact of this invasive technology that has intruded into almost every aspect of life and work has attracted significant attention from scholars in various disciplines, generating a growing body of literature on perceived risks and benefits^4–7^. A mobile device combines both communication and computing features, which can be held in hand or stored in a pocket, allowing easy access and use at the point of care^8^. Its rapidly expanding features and growing list of software applications (apps) have become deeply integrated into nearly every aspect of clinical practice, including communication between providers and patients, medical education tools, health record access, clinical decision making, and patient monitoring^2,9^.

Research on the uses of mobile devices and the impact of various apps on different aspects of healthcare continues to expand. Some of the reported advantages of mobile devices in healthcare include: allowing for more rapid decisions with lower error rates, improving quality of data management and accessibility, and improving access to evidence-based information that supports clinical decision-making at the point-of-care^10–15^. In addition, mobile devices facilitate communication among various healthcare professionals and allow for better coordination of care^9^.

However, very few studies have examined how different HCPs use mobile devices in the varying clinical care settings, both for clinical-related and for personal purposes. Although the few published studies report a high frequency of use, most of them were conducted when social media and mobile apps had yet to become as pervasive and intrusive as they are today. Nevertheless, in 2011, Smith et al. (2011) reported that 55.6% of US perfusionists acknowledged using their mobile device while performing a cardiopulmonary bypass^16^. More recently, Mobasheri et al. (2015) explored uses of mobile devices among doctors and nurses in a clinical setting in the UK, reporting high clinical-related use rates among both nurses and physicians but did not explore personal use during clinical work^17^. Katz-Sidlow et al. (2012) also looked at mobile device use during inpatient rounds and found higher self-reported usage rates among residents compared to faculty for all types of use including patient-related and non-patient care use^17,18^.

The prevalence and utilization patterns of mobile devices by HCPs in the Emergency Department (ED) has not been sufficiently studied. This setting is particularly relevant since the ED involves significant time pressures, interruptions, and complex workflows that increase safety risks. This study aims to examine the prevalence of mobile device use among attending physicians, residents, and nurses in the ED of a large academic medical center in Lebanon. The authors also aim to understand the different provider group utilization patterns and perceptions around mobile device use in the ED clinical setting as they relate to both clinical and personal use.

## Methods

### Study setting and design

The study was conducted at the ED of an academic tertiary care medical center in Lebanon, receiving an average of 57,000 visits annually. The ED includes two adult sections, a high acuity area and a low acuity area that separate patients based on the Emergency Severity Index (ESI), as well as one pediatric section for patients less than 18 years old. Each section is staffed by a team of attending physicians, residents, nurses, medical and nursing students. The ED attending physicians are a mix of American Board Certified emergency medicine physicians, and non-emergency medicine trained physicians with extensive experience in emergency care. Residents include both emergency medicine residents enrolled in a program accredited by the Accreditation Council for Graduate Medical Education International (ACGMEI), and off-service resident rotators from pediatrics, internal medicine, anesthesiology and radiology. ED nurses are all locally trained and most hold Bachelor of Nursing degrees. Medical and nursing school students also rotate in the ED among the care team. At the time the study, the ED had a team of 236 HCPs, including 32 physicians (13.6%), 53 residents (22.4%), 62 nurses (26.3%), 37 nursing students (15.7%), and 52 rotating medical students (22%).

### Measurements

For the purpose of the study, a mobile device was defined as any handheld portable network-enabled electronic device generally connected to other devices or networks via different wireless protocols^19–21^.

This study employed a cross-sectional design that utilized an online survey questionnaire to gather data from all the employees of the targeted ED. The survey questionnaire was developed in English to examine the utilization patterns of mobile devices among attending physicians, residents and nurses and determine their attitudes towards clinical and personal related use in the ED. The authors based the questionnaire on literature that examines mobile device use in clinical settings^16,22^. The authors categorized the medical and non-medical applications to capture healthcare providers’ specific use of such apps. Medical applications included applications developed specifically for clinical use including medication references/drug formularies, medical scoring systems/tools, disease diagnosis applications and procedure/documentation applications. Non-medical applications were applications that were not developed specifically for clinicians although may be used by clinicians for both clinical and personal purposes. The categories were based on previous literature examining similar topics^1,2,23^.

To enhance content validity, the questionnaire was customized to the institutional setting and reviewed by a group of experts including a statistician, an experienced ED physician, a health management and policy expert, and a communication scholar. The expert team modified the questionnaire as necessary, and a consensus was reached on the final draft, which was then piloted on a number of healthcare providers. All feedback was consolidated in the final version of the survey. Since all target HCPs were fluent in English, the questionnaire was only administered in that language.

The questionnaire included relevant demographic and utilization information (age, gender, job position, frequency of mobile device use, types of applications used, and usage types whether clinical or personal). It also included statements graded on a four-point Likert scale (“disagree”, “strongly disagree”, “strongly agree”, or “agree”) that evaluated HCPs’ attitudes toward usage of mobile devices for clinical and personal purposes in the ED.

### Data collection

Data collection was carried out between January and September 2017. All 236 healthcare providers working at the ED during the period of data collection were approached to partake in the study^24^. Those who agreed to participate were asked to click the Web-based survey link, which started with an electronic informed consent form that needed to be signed prior completing the questionnaire. Ethical approval was obtained from the Institutional Review Board (IRB) for social and behavioral studies of the American University of Beirut, under protocol number [ED.EH.06]. The research abides by the principles of scientific integrity and responsible conduct of research stipulated by the ethical principles of the Belmont Report. Prior to administering the study, participants were asked to read and sign an IRB approved electronic informed consent form emphasizing the voluntariness and anonymity aspects of the study. Participants were also informed that only aggregate data will be shared and disseminated.

### Data analysis

The data retrieved from the online questionnaire included 118 responses, out of which 18 records were discarded, as the respondents had missed more than 25% of the questions with the main scales^24^. Moreover, the data collected from nursing students (n = 3) had to be discarded since the small sample size rendered representativeness and comparability difficult^24^. To facilitate data analysis, responses were combined into two groups: agree (those who answered agree or strongly agree) and disagree (those who answered disagree or strongly disagree). SPSS 24 was used for data cleaning, management and analyses. Variables were described as number and percent for categorical variables. Associations between HCP category and each of our variables of interest were determined using the Pearson’s Chi-square test where the sample size was sufficient, and the Fisher’s Exact test where the sample size was small. We also looked at association between demographics and mobile phone usage variables. Significance was set at alpha = 0.05.

## Results

A total of 118 responses (out of 236 potential participants) were received, resulting in a 50% response rate. However, after data cleaning, only 97 responses were included in the final analysis. Demographic characteristics of our population stratified by category can be found in Table 1. Our sample was evenly divided between males and females (50.5% and 49.5%, respectively). The majority of participants (62.9%) was less than 30 years old, though this age group was mostly made up of medical students, residents and nurses. The majority of attending physicians were 30 to 39 years old (60.0%).

**Table 1:** Demographic characteristics of the target population stratified by HCP category^a, b^.

Variable		Medical student n = 23	Nurse n = 32	Resident n = 22	Attending physician n = 20	Total	*P* value
Gender	Male	10 (50.0)	14 (43.8)	9 (40.9)	14 (73.7)	47 (50.5)	0.14
	Female	10 (50.0)	18 (56.3)	13 (59.1)	5 (26.3)	46 (49.5)	
Age	< 30	23 (100.0)	19 (59.4)	19 (86.4)	0 (0.0)	61 (62.9)	< 0.0001
	30–39	0 (0.0)	8 (25.0)	3 (13.6)	12 (60.0)	23 (23.7)	
	≥ 40	0 (0.0)	5 (15.6)	0 (0.0)	8 (40.0)	13 (13.4)	

^a^Cell counts may not add up to the raw total due to missing responses in the data.

^b^Valid percentages were used.

Table 2 exhibits the providers’ mobile device use, stratified by category. Analysis reveals that 100% of survey respondents owned a mobile device (a smart phone in particular) and brought their mobile device to their ED shift. A significant difference appears across HCP categories for owning a tablet (52.2%, 9.4%, 40.9% and 20.0% among medical students, nurses, residents and attending physicians, respectively, *P* = 0.002). The proportion of people who had medical apps on their mobile devices was much higher among residents and attending physicians (100.0% and 90.0%, respectively) than among medical students and nurses (82.6% and 75.0%, respectively, *P* = 0.05). Mobile device use in the ED was comparable across categories, with 91.3% of medical students, 90.6% of nurses, 95.5% of residents and 90.0% of attending physicians reporting that they use their mobile devices for clinical care-related purposes. Regarding the use of mobile devices for personal reasons, 82.6% of medical students, 83.9% of nurses, 77.3% of residents and 75.0% of attending physicians reported using their mobile devices during their ED shift for non-work related matters, such as online banking and personal messaging.

**Table 2:** Healthcare providers’ self-reported mobile device use stratified by HCP category ^a, b^.

Variable		Total n = 97	Medical student n = 23	Nurse n = 32	Resident n = 22	Attending physician n = 20	*P* value
Own a mobile device	Yes	97 (100.0)	23 (100.0)	32 (100.0)	22 (100.0)	20 (100.0)	*
Type of mobile device owned	A smart phone	97 (100.0)	23 (100.0)	32 (100.0)	22 (100.0)	20 (100.0)	*
	Tablet	28 (28.9)	12 (52.2)	3 (9.4)	9 (40.9)	4 (20.0)	0.002
	Smart watch/ band	3 (3.1)	1 (4.3)	0 (0.0)	1 (4.5)	1 (5.0)	0.54
Do you have any medical applications on your mobile device?	Yes	83 (85.6)	19 (82.6)	24 (75.0)	22 (100.0)	18 (90.0)	0.05
Bring mobile device to your ED shift	Yes	97 (100.0)	23 (100.0)	32 (100.0)	22 (100.0)	20 (100.0)	*
Do you use your mobile device in the ED for clinical care related use (HIS/UpToDate/ drug references…etc.)?	Yes	89 (91.8)	21 (91.3)	29 (90.6)	21 (95.5)	18 (90.0)	0.92
Do you use your mobile device for personal reasons during your shift in the ED? (E.g. web surfing/WhatsApp/Online games/banking)?	Yes	77 (80.2)	19 (82.6)	26 (83.9)	17 (77.3)	15 (75.0)	0.86

*Indicates that a *P* value could not be obtained due to uniformity in the responses.

^a^Cell counts may not add up to the raw total due to missing responses in the data.

^b^Valid percentages were used.

The use of non-medical apps was very common, but the type of apps used differed among specialists (Table 3). Medical students used their mobile devices mainly to access their email (91.3%) and to communicate via messaging applications (91.3%). Medical Students also exhibited greater use of social media apps (87.0%), compared to nurses (66.7%), residents (63.6%) and attending physicians (45.0%). Attending physicians equally used their mobile devices to make phone calls (90.0%), access their emails (90.0%), browse the web (90.0%), and to communicate via messaging applications (90.0%). Nurses used their mobile devices mainly to access their email (93.6%) and browse the web (87.5%).

**Table 3:** Most used medical and non-medical applications stratified by HCP category^a, b, c^.

Variable		Total	Medical student n = 23	Nurse n = 32	Resident n = 22	Attending physician n = 20	*P* value
Most used medical applications	Medication formulary/drug reference apps	81 (84.4)	19 (82.6)	25 (78.1)	21 (100.0)	16 (80.0)	0.10
	Medical scoring systems/calculators	64 (66.7)	14 (60.9)	18 (58.1)	18 (81.8)	14 (70.0)	0.29
	Disease diagnosis/management apps	66 (69.5)	16 (72.7)	19 (61.3)	19 (86.4)	12 (60.0)	0.18
	Procedure documentation apps	22 (26.2)	2 (9.1)	7 (33.3)	9 (42.9)	4 (20.0)	0.06
Most used non-medical applications	Email access	86 (91.5)	21 (91.3)	29 (93.6)	18 (90.0)	18 (90.0)	0.92
	Messaging applications	84 (87.5)	21 (91.3)	25 (80.7)	20 (90.9)	18 (90.0)	0.67
	Web access	81 (84.4)	20 (87.0)	28 (87.5)	15 (71.4)	18 (90.0)	0.38
	Phone calls	73 (76.0)	16 (69.6)	23 (74.2)	16 (72.7)	18 (90.0)	0.41
	Calendar	72 (75.8)	17 (73.9)	25 (80.7)	14 (66.7)	16 (80.0)	0.69
	Social media applications	63 (66.3)	20 (87.0)	20 (66.7)	14 (63.6)	9 (45.0)	0.03

^a^Cell counts may not add up to the raw total due to missing responses in the data.

^b^Valid percentages were used.

^c^Answers are not mutually exclusive, each respondent chose all that were applicable.

Moreover, the most popular medical applications used by residents, medical students, attending physicians and nurses are medical formulary/drug reference apps (100.0%, 82.6%, 80.0%, and 78.1%, respectively), followed by disease diagnosis/management apps (86.4%, 72.7%, 60.0%, and 61.3%, respectively) and medical scoring systems/calculator (81.8%, 60.9%, 70.0% and 58.1%, respectively). Procedure documentation apps were used the least, mainly by residents (42.9%).

Table 4 shows provider attitudes towards mobile devices, stratified by HCP category. The majority agreed that mobile devices enabled better coordinated care among providers across the hospital (76.5%, 78.1%, 83.3% and 90.0% among attending physicians, nurses, medical students and residents, respectively) and that mobile device use is beneficial to patient care (90.0%, 74.2%, 90.5% and 61.1%, respectively). Nurses registered a higher level of agreement (71.0%) in regard to mobile device use improving ED cohesion and teamwork, compared to medical students, residents and attending physicians (52.6%, 47.4% and 37.5%, respectively). Less than half of the attending physicians felt that mobile device use by providers improves patient safety (44.4%), in comparison to more than half of the students, nurses and residents (55.6%, 53.3% and 82.4%, respectively). Most of the respondents agreed that mobile device use assisted in quickly resolving personal issues (85.0%, 86.7%, 81.0% and 61.1% among students, nurses, residents and attending physicians, respectively) and that using mobile devices reduces their feeling of stress at work (79.0%, 77.4%, 84.2% and 55.6%, respectively). On the other hand, few respondents agreed with the statement that using mobile devices for non-work related purposes positively impacts their performance at work (36.4%, 50.0%, 35.0% and 25.0% among students, nurses, residents and attending physicians, respectively).

**Table 4:** Personal and professional attitudes of HCPs towards mobile device use stratified by category^a^.

Variable		Total n = 97	Medical student n = 23	Nurse n = 32	Resident n = 22	Attending MD n = 20	*P* value
Mobile device use enabled better coordinated care among the medical providers across the hospital	Agree	71 (81.6)	15 (83.3)	25 (78.1)	18 (90.0)	13 (76.5)	0.68
	Disagree	16 (18.4)	3 (16.7)	7 (21.9)	2 (10.0)	4 (23.5)	
Mobile device use improved ED cohesion and teamwork	Agree	47 (55.3)	10 (52.6)	22 (71.0)	9 (47.4)	6 (37.5)	0.13
	Disagree	38 (44.7)	9 (47.4)	9 (29.0)	10 (52.6)	10 (62.5)	
Mobile device use improved patient safety	Agree	48 (57.8)	10 (55.6)	16 (53.3)	14 (82.4)	8 (44.4)	0.12
	Disagree	35 (42.2)	8 (44.4)	14 (46.7)	3 (17.7)	10 (55.6)	
Mobile device use is beneficial to patient care	Agree	71 (78.9)	18 (90.0)	23 (74.2)	19 (90.5)	11 (61.1)	0.08
	Disagree	19 (21.1)	2 (10.0)	8 (25.8)	2 (9.5)	7 (38.9)	
Mobile device use assisted me in resolving my personal issues quickly, and thus improves my ability to focus on my work	Agree	71 (79.8)	17 (85.0)	26 (86.7)	17 (81.0)	11 (61.1)	0.20
	Disagree	18 (20.2)	3 (15.0)	4 (13.3)	4 (19.1)	7 (38.9)	
Using my mobile device for non-work related purposes has positively impacted my performance at work	Agree	34 (38.6)	8 (36.4)	15 (50.0)	7 (35.0)	4 (25.0)	0.38
	Disagree	54 (61.4)	14 (63.6)	15 (50.0)	13 (65.0)	12 (75.0)	
Mobile device use reduced my stress at work	Agree	65 (74.7)	15 (79.0)	24 (77.4)	16 (84.2)	10 (55.6)	0.23
	Disagree	22 (25.3)	4 (21.1)	7 (22.6)	3 (15.8)	8 (44.4)	

^a^Cell counts may not add up to the raw total due to missing responses in the data.

## Discussion

This study is the first to focus on mobile device utilization in the ED setting and to explore both HCP clinical-related and personal use in this setting. The study revealed that 100% of HCP owned a mobile device, irrespective of their category, and that the majority use their mobile devices for clinical-related and personal purposes during their shifts. Findings of this study show even higher ownership and use rates than previously published studies, which had already documented increasing trends^1,17,18,25^. These studies were published more than 4 years ago, a relatively long period during which mobile device accessibility, internet speed, mobile social media penetration, and the prevalence of mobile applications advanced at neck-break speed. In fact, global penetration of mobile devices increased from 56.5% in 2013 to 67.1% in 2019^26^; mobile device internet user penetration grew from 48.8% in 2014 to 63.4% in 2019^27^; mobile device social media penetration more than doubled between 2014 and 2018^28^ worldwide, while the reach of 4G networks has continued to expand worldwide.

### Healthcare use

Results of this study demonstrate high use of medical applications among all HCPs, with resident use being the highest for all types of applications, similar to what other studies have reported^16,17,25^. Medical formulary/drug referencing applications were the most commonly used applications by all ED HCPs, followed by disease diagnosis/management applications, likely reflecting the increased complexity of current care and growing need of HCP for tools to assist them in integrating the rapidly evolving literature on up to date clinical management. Similar to results of other studies, while nurses used drug formulary applications the most, overall usage of medical applications in general was lower for nurses than other groups. This may be because available medical applications are targeting the needs of physicians more than nurses. This area needs further exploration as it raises the possibility of unexplored potential mobile device uses targeting nurses’ needs.

### Attitudes to health care use

Study findings further reveal an overwhelmingly positive attitude by all HCP categories towards mobile device use for coordination of patient care which has been supported by prior studies that looked at coordination of care and communication among HCP^2,29^. However, personal use of such devices may lead to distraction that may negatively affect team cohesion and the quality of patient experience and care. This may possibly explain the less favorable attitudes expressed in this study, especially by attending physicians, toward impact on patient safety and ED teamwork and cohesion. It may also be related to a generational effect, where attending physicians, who are relatively older compared to the other professional groups, may be less friendly towards technology^25,30^ and may have less experience with mobile devices than their younger counterparts, as well as greater concerns over privacy and security^31–34^. Health care managers need to investigate and address generational gaps, as they might affect the work dynamics among HCP and, as such, may require regulation to curb the potential negative impact of mobile device use on teamwork.

### Personal use

Our study further reveals that mobile device use by HCPs in clinical settings was not limited to clinical care but rather extended to extensive personal use. In fact, an overwhelming majority of all responding HCPs reported using their mobile devices in the ED for personal reasons, with attending physicians reporting the lowest personal use (75%). While all HCP categories reported high email and messaging application use, phone (voice) use was highest among attending physicians, and social media use was highest among medical students. In fact, our findings reveal higher social media use in the clinical areas compared to prior studies^16^. Use of phone (voice) by attendings may again reflect a generational effect, with the relatively older attending physicians more accustomed to using the mobile devices, particularly phones, for making calls (similar findings have been reported in the UK by Mobasheri et al., 2015). On the other hand, younger HCPs are more likely to use mobile devices for social media communications. This may also be explained by older respondents being more aware of, and compliant with, the safety standards associated with using social media applications^16^, but global trends show that the younger people, especially those under 34 years old, exhibit significantly higher social media use^35^. Irrespective of the reported type of use, the high rates of personal use of mobile devices in our study are disconcerting particularly in an ED setting, where there is a growing body of literature around the impact of interruptions and distraction on patient safety^24^. The fact that younger generations have reported higher rates of difficulty in giving up social media^36^ makes the problem more concerning for that age group, which will staff the future ED.

### Attitude towards personal use

Although four out of five respondents stated that mobile device use improves their ability to focus on their work by helping them resolve personal issues, the majority disagreed (61.4%) that using mobile devices for non-work related purposes positively impacts their performance at work, and many felt that mobile devices do not reduce their stress level at work. These findings may reflect a reverse “spillover” effect of mobile device on worktime. Although much has been written about work spillover on personal time, what our study shows is the intrusion of personal time/issues on work through mobile devices. This is an area that deserves further exploration especially in light of the already documented association between home-work interference and increased burnout which ultimately leads to a decrease in job satisfaction and organizational commitment^37–39^.

### Regulation implications

Although mobile device use has become integral to clinical care, there is growing evidence for the need for some regulation of personal use in the clinical setting. Mobile devices, however, are highly complex technologies that seamlessly integrate a myriad of media platforms and services, both professional and personal, making it exceedingly difficult to manage their risks and benefits in the workplace. Their multiservice aspect renders the historical professional/private divide almost obsolete. Moreover, historically the regulation, control and even banning of technology has not been effective or even practical modes of regulation^40^. In addition, restrictions and bans have been found counterproductive, as they eliminate the beneficial use of mobile devices^41^. Therefore, it is critical to also include technological solutions, or what Levinson^40^ calls “remedial media.” We can imagine institutional apps that control or limit certain mobile device features during certain peak times or risky spaces^41^. In addition, recent studies highly recommend institutionalizing a digital code of conduct that would ensure the safe use of mobile devices in health care settings^42^.

### Methodological approaches

While the authors considered using an ethnographic observational study, this was ultimately decided against due to the intrusiveness of the approach for both patients and providers in an acute care setting. In addition, concerns about changes of behavior from on-site observation may impact provider behavior as well as reporting. As for use of screen time applications to assess provider usage, while this would certainly yield interesting data, it unfortunately was not commonly available on most smartphones at the time of the study in 2017. Most of the built-in screen time tracking capabilities were implemented for IOS users in 2018. Furthermore, these devices would not allow investigators to assess whether specific applications are being used for clinical care or personal use. For example, Wi-Fi messaging systems (such as WhatsApp) are used for communication between consulting teams in our setting and can also be used for personal messaging.

The momentary survey method was not considered and may have been better able to capture the usage frequency and patterns of smart devices by ED providers. However, it would be quite challenging to convince busy ED providers to report on their experience several times a day, and this would have significantly reduced the response rate.

### Limitations

The results of our study should be considered in the light of its limitations. First, the study utilized a cross-sectional survey approach and relied on health care providers’ subjective reporting of SD usage. This approach could lead to inaccurate reporting when compared with other approaches (e.g. usage monitoring applications). Having said that, the authors believe that the approach was the most suitable for the context of this study, since it provided balanced considerations of privacy, response rate, validity in tracking personal vs professional usage, and feasibility of implementation in a busy ED setting. Second, although the research team assured healthcare providers with the confidentiality of their responses, there remains a risk of a social desirability bias with respondents potentially modifying their responses to reflect positive mobile device use. Third, the cross-sectional nature of the study only allows the establishment of associations rather than causal relationships. In addition, the nature of the study being single centered represents a limitation. However, the targeted hospital is one of the largest tertiary care academic centers in Lebanon, therefore increasing the generalizability of our results to other healthcare institutions. Fourth, the small sample size might have influenced the power required to reveal significant differences. Last, the study had a 50% response rate which, despite being an acceptable rate for online questionnaires, especially in healthcare settings, has missed the responses of half of the target population, who may have different opinions.

## Conclusion

The use of mobile devices in clinical settings is the new occupational reality. The ED setting is no exception despite its heavy workloads and tendency for interruptions. While ED HCPs seem to value the impact of mobile devices on coordination of care, attitudes towards impact on safety and team cohesion are less favorable. In addition, the reverse spillover effect of personal issues into the workplace enabled by mobile devices may have some negative impact on staff’s performance at work. It is imperative to engage HCPs, patients and concerned stakeholders in the formulation of a contextual regulatory framework that may include technologic restrictions as well as a digital code of conduct that can be adopted in the health care setting.
